# FGF19 promotes nasopharyngeal carcinoma progression by inducing angiogenesis via inhibiting TRIM21-mediated ANXA2 ubiquitination

**DOI:** 10.1007/s13402-023-00868-9

**Published:** 2023-10-02

**Authors:** Si Shi, Qicheng Zhang, Kaiwen Zhang, Wenhui Chen, Haijing Xie, Si Pan, Ziyi Xue, Bo You, Jianmei Zhao, Yiwen You

**Affiliations:** 1 Department of Otorhinolaryngology Head and Neck Surgery, Affiliated Hospital of Nantong University, Medical School of Nantong University, Nantong, 226001 Jiangsu Province China; 2 Institute of Otorhinolaryngology Head and Neck Surgery, Affiliated Hospital of Nantong University, Nantong, 226001 Jiangsu Province China; 3 Department of Paediatrics, Affiliated Hospital of Nantong University, Nantong, 226001 Jiangsu Province China

**Keywords:** Nasopharyngeal carcinoma, FGF19, Angiogenesis, ANXA2, TRIM21

## Abstract

**Purpose:**

Nasopharyngeal carcinoma (NPC) has characteristics of high invasion and early metastasis. Most NPC patients present with locoregionally advanced illness when first diagnosed. Therefore, it is urgent to discover NPC biomarkers. Fibroblast growth Factor 19 (FGF19) plays a role in various physiological or pathological processes, including cancer. In this research, we discovered the importance of FGF19 in NPC, and clarified its role in tumour angiogenesis.

**Methods:**

Western blotting, immunohistochemistry and ELISA were used to investigate FGF19 expression in NPC. Then we took CCK8, colony formation, Transwell and wound healing assays to identify the influence of FGF19 on NPC malignant behaviours. The proliferative and metastatic capacity of FGF19 were evaluated in nude mice and zebrafish. The role of FGF19 in angiogenesis was investigated by tube formation and Matrigel plug angiogenesis assays. We then evaluated the variation in Annexin A2(ANXA2) levels with the treatment of FGF19. Lastly, co-immunoprecipitation and ubiquitination assays were performed to identify the mechanisms involved.

**Results:**

FGF19 levels were elevated in tissues and serum of NPC patients and were associated with poor clinical stages. High expression of FGF19 promoted NPC malignant behaviours. In particular, FGF19 expression was correlated with microvessel density in tissues and NPC-derived FGF19 could accelerate angiogenesis in vitro and in vivo. Mechanistically, FGF19 influenced ANXA2 expression to promote angiogenesis. Moreover, tripartite motif-containing 21(TRIM21) interacted with ANXA2 and was responsible for ANXA2 ubiquitination.

**Conclusion:**

FGF19 promoted NPC angiogenesis by inhibiting TRIM21-mediated ANXA2 ubiquitination. It may serve as a noninvasive biomarker for NPC and provides new insights for therapy.

**Supplementary Information:**

The online version contains supplementary material available at 10.1007/s13402-023-00868-9.

## Introduction

Nasopharyngeal carcinoma (NPC), an epithelial carcinoma derived from the mucosal lining, has a distinct global geographic distribution. According to the International Agency for Research on Cancer, there were over 130,000 new cases of NPC reported in 2020. More than 70% of those new cases were distributed in east and southeast Asia [1, 2]. With advances in radiotherapy and chemotherapy, the mortality rate of NPC has shown a downwards trend in recent years [3]. However, given the deep-tissue location and concealed early symptoms, with characteristics of high invasion and early metastasis, more than 70% of patients present with locoregionally advanced illness when they are diagnosed, which might lead to poor prognosis [4, 5]. Therefore, identifying biomarkers specific to NPC might help to guide early diagnosis and new therapeutic methods.

To manage the rapid growth of malignant cells, tumours develop a new vascular network [6, 7]. New vessels transport oxygen and nutrients for tumour survival, and are one of the vital hallmarks of metastasis [8, 9]. During tumour development, pro- and antiangiogenic modulators are imbalanced, and the “angiogenic switch” is turned on [10]. The newly formed tumour vascular network can accelerate tumour growth and metastasis [11, 12]. It has been reported that tumour cells or stromal cells can secrete abnormal levels of proangiogenic factors and contribute to the creation of a tumour vascular network [6]. In our previous research, we found that tumour-derived factors could promote NPC progression [13–15]. Additionally, antiangiogenic therapies could correct tumour vessel function and make tumours less aggressive [16, 17]. However, current antiangiogenic therapies have not achieved these ideal effects. Thus, it is urgent to explore the molecular mechanism of NPC angiogenesis.

The fibroblast growth factor (FGF) family is composed of 22 structurally related polypeptides with different biological activities [18]. Most FGFs bind to and activate cell surface FGF receptors to exert their functions in physiological or pathological processes, including embryonic development, tissue regeneration and tumour progression [19, 20]. Some FGFs might act as proangiogenic factors, activating signal transduction to induce angiogenic responses [21].

FGFs are divided into seven subfamilies. Among them, a newly discovered group of noncanonical, endocrine-like FGFs has attracted much attention in recent years [22]. This subfamily includes FGF19, its murine homologue FGF15, FGF21 and FGF23, which can be secreted and function as endocrine factors or hormones [23, 24]. FGF19 is a highly conserved gene and has unique functions in different tissues [25]. Under physiological conditions, FGF19 acts as a growth factor and regulates lipid, protein and glucose metabolism during organogenesis [20, 22]. FGF19 also participates in cell proliferation, differentiation, and motility in certain pathologies [26]. Aberrant activation of FGF19 might contribute to neoplasia development [27]. It is overexpressed in a variety of tumours and serves as an oncogenic driver [28–31]. In our previous research, we found that FGFR4, the receptor of FGF19, was upregulated in clinical NPC samples and associated with poor prognosis [32]. However, the role of FGF19 in NPC has not been elucidated.

In this research, we reported the functional role of FGF19 in NPC development. It was highly expressed in the tissues and serum of NPC patients and could promote a malignant tumour phenotype. In particular, we found that NPC-derived FGF19 could enhance tumour angiogenesis. Mechanistically, FGF19 decreased Annexin A2(ANXA2) degradation by influencing tripartite motif-containing 21(TRIM21)-mediated ANXA2 ubiquitination, leading to the promotion of angiogenesis. Our research confirmed the importance of FGF19 in NPC and might provide new insight for therapy.

## Materials and methods

### Patients and immunohistochemistry

Paraffin-embedded NPC specimens were retrieved from the Department of Pathology, Affiliated Hospital of Nantong University. Noncancerous samples in the nasopharynx were used as controls. Fresh tissues and serum samples were obtained from the Department of Otorhinolaryngology Head and Neck surgery，Affiliated Hospital of Nantong University. The clinical processes were approved by the Ethics Committee of Affiliated Hospital of Nantong University (IRB number: 2017-L080), and each patient provided prior consent.

Immunohistochemistry was carried out according to the general procedures previously described [14]. To quantify FGF19 expression, we multiplied the scores of the staining intensity and the percentage of positive cells to define the final score. The intensity of FGF19 staining was scored as 1, no staining; 2, weak staining; 3, medium staining; 4, strong staining. The percentage of positive cells was scored as 1 ≤ 25; 2, 26–50%; 3, 51–75%; 4 > 75%.

Microvessel density (MVD) was evaluated as described by Foote and our previous research [14, 33]. Briefly, to quantify the vessel density, we divided the xenograft into three layers and cutting three tissue slices from each layer. Tumour sections were scanned at a low power and we found areas with high MVDs (brown staining). Individual microvessels were counted in three fields under a magnification of 200 × field. The primary antibodies used included anti-FGF19 (sc-390621, Santa Cruz Biotechnology, Texas, USA,1:50), anti-CD34 (14486–1-AP, Proteintech, China,1:100) and anti-Ki67 (27309–1-AP, Proteintech, China, 1:2000).

### Cell culture

The NPC cell lines CNE1(high differentiation), CNE2(low differentiation), 5-8F (high tumorigenesis and high metastasis), 6-10B (high tumorigenesis and low metastasis), and C666-1 (EBV + and low differentiation) and the immortalized normal nasopharyngeal epithelial cell line NP69 were kindly provided by the Sun Yat-Sen University and Xiang-Ya School of Medicine. Human umbilical vein endothelial cells (HUVECs) were cultured in DMEM/F-12 (HAM) 1:1 (C3130, Viva Cell Biosciences, China) containing 10% FBS (04–001-1ACS, Biological Industries, Beit-Haemek, Israel). Tumour cells were grown in RPMI 1640 (C3010, Viva Cell Biosciences, China) with 10% FBS, while NP69 cells were maintained in Keratinocyte-SFM medium (10785, Gibco, Grand Island, USA). Cells were cultured at 37 °C under 5% CO_2_ at the Institute of Otorhinolaryngology Head and Neck Surgery, Affiliated Hospital of Nantong University (Jiangsu, China).

### ELISA analysis of FGF19 expression

We collected the cell culture supernatant and centrifuged it to remove particulates. Serum was collected with a serum separator tube and centrifuged for 15 min at 1000 g. A human FGF19 immunoassay ELISA kit (Quantikine® ELISA DF1900, R&D Systems, Minnesota, USA) was used to measure FGF19 levels in serum and culture medium according to the manufacturer’s protocol. The optical density of each well was determined with a microplate reader at 450 nm.

### Western blot

Western blotting was performed as previously described [32].The antibodies used were as follows: anti-FGF19 (A6589, ABclonal, China, 1:500), anti-TRIM21 (12108–1-AP, Proteintech, China, 1:1000), anti-ANXA2 (sc-28385, Santa Cruz Biotechnology, Texas, USA, 1:1000), anti-Tubulin(FD0064, Fude bio, China, 1:1000), anti-p-PI3K (A0982, ABclonal, China, 1:500), anti-AKT1 (A11016, ABclonal, China, 1:1000), anti-p-AKT1 (AP0140, ABclonal, China, 1:1000), anti-mTOR (A11355, ABclonal, China, 1:500), and anti-p-mTOR (ab109268, Abcam, UK, 1:1000).

### Quantitative RT-PCR

Total RNA was extracted from cells and tissues using Trizol (M5100, New Cell& Molecular Biotech, China) according to the manufacturer’s instructions. cDNA was generated by a reverse transcriptase kit (K1622, Thermo Fisher Scientific, USA). Then qRT-PCR was performed with a SYBR Green Master (04913914001, Roche, Germany) in a Real-Time PCR System. Gene expression level was analyzed using the ΔΔCt method and normalized to GAPDH expression. Primers were obtained from Sangon Biotech(China) and listed as follows: FGF19: Forward: 5'- ATGGCTACAATGTGTACCGATC-3', Reverse: 5'- AGAAAGCCTCTGTTCTTGTACA-3'; TRIM21: Forward: 5'- TCCTTCTACAACATCACTGACC-3', Reverse: 5'- CAATATTCAGTGGACAGAGGGT-3'; ANXA2: Forward: 5`- ACATTGAAACAGCCATCAAGAC-3', Reverse: 5`- GAAGGCAATATCCTGTCTCTGT -3'.

### Transfection with plasmids and siRNAs

Cell transfection was performed according to the manufacturer’s instructions. Three short hairpin RNAs (shRNAs) against FGF19 or ANXA2 and FGF19 overexpression plasmid were constructed by GeneChem Co., Ltd. (China). Small interfering RNAs (siRNAs) targeting TRIM21 were obtained from Tsingke Biotechnology Co., Ltd.(China). The sequences are listed in Table1.

**Table 1:** Sequences of siRNAs and shRNAs

Name	Sequences(5’-3’)
TRIM21-siRNA1	Forward: 5'-GCAUGGUCUCCUUCUACAATT-3' Reverse: 5'-UUGUAGAAGGAGACCAUGCTT-3'
TRIM21-siRNA2	Forward: 5'-GGUGAUAAUUGUCCUGGAATT-3' Reverse: 5'-UUCCAGGACAAUUAUCACCTT-3'
TRIM21-siRNA3	Forward: 5'-CGCAGAGUUUGUGCAGCAATT-3' Reverse: 5'-UUGCUGCACAAACUCUGCGTT-3'
FGF19-shRNA1	forward sequence 5′-CCGGCCACTTGGAATCTGACATGTTCTCG AGAACATGTCAGATTCCAAGTGGTTTTTG-3′
FGF19-shRNA2	forward sequence 5′-CCGGGCTTTCTTCCACTCTCTCATTCTCG AGAATGAGAGAGTGGAAGAAAGCTTTTTG-3′
FGF19-shRNA3	forward sequence 5′-CCGGCAATGTGTACCGATCCGAGAACTC GAGTTCTCGGATCGGTACACATTGTTTTTG-3′
ANXA2-shRNA1	forward sequence 5′-CCGGCTGTACTATTATATCCAGCAACTC GAGTTGCTGGATATAATAGTACAGTTTTTG-3′
ANXA2-shRNA2	forward sequence 5′-CCGGCCTGCTTTCAACTGAATTGTTCTCG AGAACAATTCAGTTGAAAGCAGGTTTTTG-3′
ANXA2-shRNA3	forward sequence 5′-CCGGTGAGGGTGACGTTAGCATTACCTC GAGGTAATGCTAACGTCACCCTCATTTTTG-3′

### Immunofluorescence assay

HUVECs were seeded onto slides overnight and fixed with 4% paraformaldehyde. Then, they were blocked for 1 h and incubated with anti-TRIM21 (12108–1-AP, Proteintech, China, 1:50) and anti-ANXA2 (sc-28385, Santa Cruz Biotechnology, Texas, USA, 1:50) antibodies. The next day, the cells were incubated with a fluorescent dye-labelled secondary antibody for 1 h. We washed the slides and added an anti-fluorescence attenuator containing DAPI. Images of stained cells were captured under a confocal microscopy. Pearson's R value of scatter plot analysis was calculated using ImageJ.

### Cell proliferation assay

Cells were seeded onto 96-well plates(Corning, USA) at a density of 5 × 10^3^ cells per well. Cell viability was assessed using CCK-8 (BS350B, Biosharp Life Sciences, China). The absorbance of each well was measured by a microplate reader.

### Colony formation

Five hundred cells were seeded on a 6-well plate and incubated for 10 days. Then, they were fixed and stained with crystal violet. Colonies that contained more than 50 cells were counted as one positive colony.

### Wound healing assay

Cells were seeded on a 6-well plate to reach 80% confluence. A single scratch wound was created with a 100-μl pipette tip. Then, serum-free medium was added to replace the culture medium. Every 12 h, we observed the migration distance of the cells. Relative distance was measured by the wound width/the distance measured at 0 h.

### Transwell migration assay

A total of 5 × 10^4^ cells were resuspended in 200 μl serum-free medium and added to the upper chamber with a polycarbonate filter of 8 μm. Then, complete medium was added to the lower chamber. After 18 h (for NPC cells) or 12 h (for HUVECs) of incubation, cells that had passed through the membrane were fixed and stained. Migrated cells were counted under a microscope in 5 random selected fields.

### Tube formation assay

96-well plate was coated with 50 μL of Matrigel (Matrigel Matrix 354234, Corning, USA) and incubated at 37 °C for 30 min. Then, 3 × 10^4^ HUVECs were resuspended in 100 μl serum-free medium and seeded into the precoated well for 6 h. Images of the capillary-like structures were captured. Relative tube length was calculated by ImageJ.

### Matrigel plug assay

HUVECs** (**3 × 10^6^) were collected and mixed with 300 μl Matrigel. Then, the mixture was subcutaneously injected into five-week-old BALB/c male nude mice. One week later, the plugs were obtained and processed into frozen sections. We stained the sections with haematoxylin and eosin to observe the vessels.

### Coimmunoprecipitation

For Co-IP, we used a Pierce Co-Immunoprecipitation Kit (26149, Thermo Fisher Scientific, USA) according to the manufacturer’s instructions. Proteins were immunoprecipitated with antibodies and then subjected to western blotting.

### Cycloheximide chase assay

The stability of ANXA2 was determined by a cycloheximide (CHX) chase assay. HUVECs cells were seeded on 6-well plates and treated with different stimulations for 24 h. Then, 100 µg/ml CHX (Med Chem Express, New Jersey, USA) was added. At different time points, cells were collected for western blotting.

### Ubiquitylation assay

To analyse ANXA2 ubiquitination, the proteasome inhibitor MG132(20 μM) (A2585, APExBIO, Houston, USA) was added to HUVECs and incubated for 6 h. Then, lysates were collected and immunoprecipitated with anti-ANXA2. Western blotting was performed to determine ubiquitylation levels with anti-ubiquitin (ab134953, Abcam, UK, 1:1000).

### In vivo experiments

CNE2 and C666-1 cells were transfected with shFGF19 or shNC and then subcutaneously injected into 5-week-old BALB/c male nude mice. Tumours were measured every two days and the volume was calculated as 1/2 (length (mm)) × (width (mm))^2^. Two weeks later, the xenografts were excised, fixed and embedded in paraffin for subsequent IHC assays. All experiments were performed following the NIH guidelines and were approved by the Animal Experiments Ethics Committee of Nantong University (Ethics number: 20170309–001).

### Zebrafish experiments

We used *Tg (fli1a: EGFP)* transgenic zebrafish for the tumour migration assay. Approximately 300 cells that had been stained with DiI (C1036, Beyotime, China) were injected into the perivitelline cavity of 48 h postfertilization zebrafish embryos with a microinjection system. DiI-stained cells were visualized under a fluorescence microscope.

For angiogenesis assays in zebrafish, the FGF19 overexpression plasmid was injected into 1–2-cell stage fertilized eggs of *Tg (fli1a: EGFP)* transgenic zebrafish. Seventy-two hours later, we observed the morphology of subintestinal vessels (SIVs) with a confocal microscopy (TCS-SP5 LSM, Leica, Germany).

### Statistical analysis

Data were collected from three independent experiments and expressed as the mean ± standard deviation (SD). Statistical analysis was performed by SPSS17.0 software and GraphPad Prism. Comparisons between different groups were analysed using Student’s t test and one-way ANOVA. Correlation analysis was performed using Spearman’s rank correlation coefficient. The following *P* values were denoted as statistical significance: *P < 0.05, **P < 0.01, ***P < 0.001.

## Results

### FGF19 is highly expressed in NPC

**Fig. 1:**
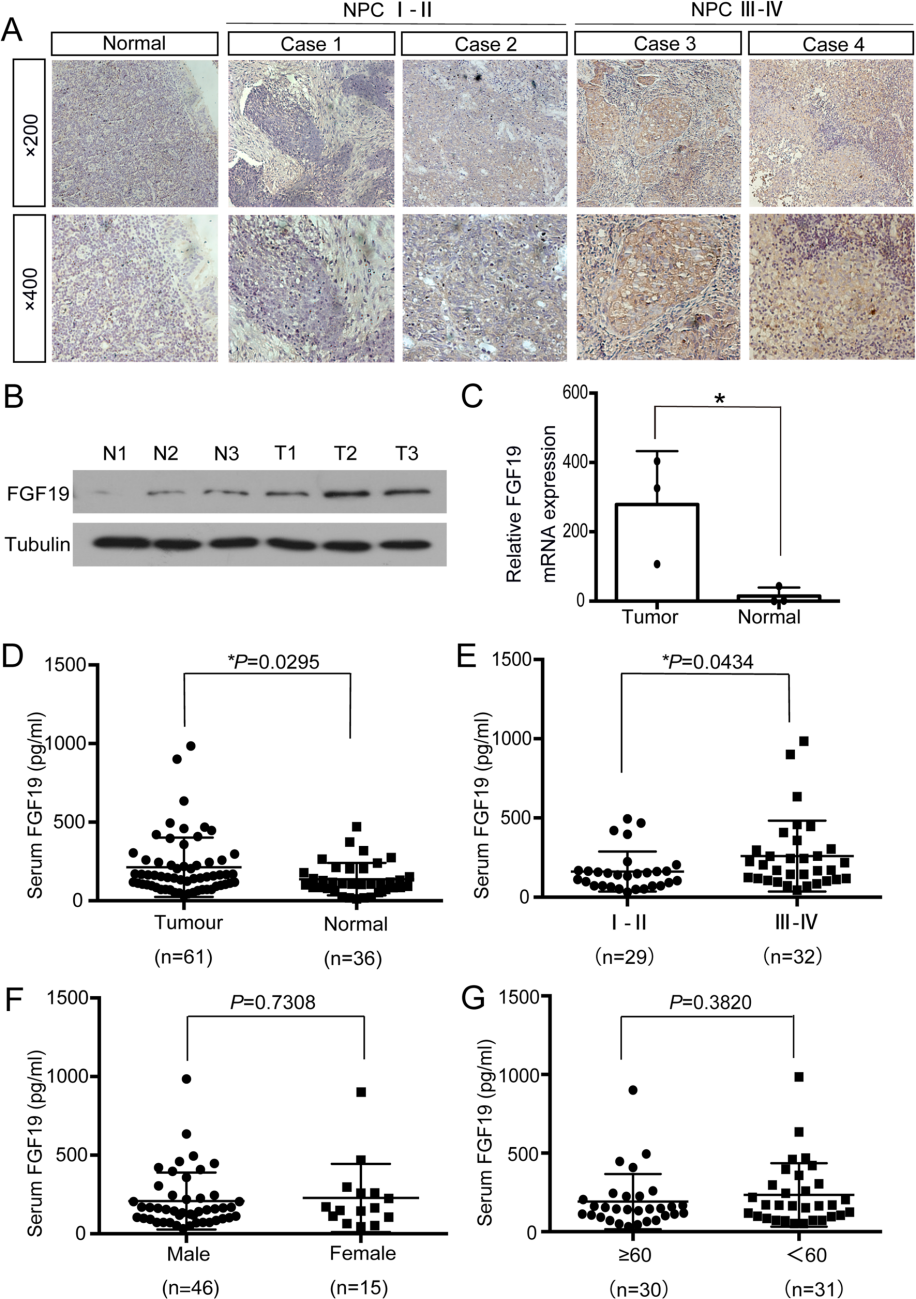
FGF19 is highly expressed in NPC. A: Representative results of immunohistochemical staining. The first column: IHC detection of FGF19 in nasopharyngeal epithelium tissues. The second and third columns: IHC detection of FGF19 in NPC tissues of stage I-II. The forth and fifth columns: IHC detection of FGF19 in NPC tissues of stage III-IV (top: × 200, bottom: × 400). B: Western blot analysis of FGF19 expression in 3 NPC tissues and 3 nasopharyngeal epithelium tissues. (T) Nasopharyngeal squamous cell carcinoma tissues. (N) Nasopharyngeal epithelium tissues. Tubulin was used as a control for protein load. C: qRT-PCR was used to detect the relative expression of FGF19 in tissues. D: ELISA was used to detect serum FGF19 levels in 61 NPC patients and 36 healthy volunteers. E: Serum FGF19 levels of NPC patients in stage I- II and stage III- IV. F: Serum FGF19 levels in male and female NPC patients. G: Serum FGF19 levels in NPC patients of different ages. Data are presented as the mean ± SD of three independent assessments. *P < 0.05, **P < 0.01, ***P < 0.001, NS: nonsignificant

To identify the role of FGF19 in NPC, we first analysed its expression level in tissues. Immunohistochemistry (IHC) and western blotting demonstrated that most NPC samples had abnormally positive FGF19 immunoreactivity, while in nasopharyngeal epithelium tissues, FGF19 was downregulated or absent (Fig. 1A-B). More importantly, from the IHC results, we found that FGF19 was more highly expressed in patients in stages III- IV than in patients in stage I- II (Fig. 1A). qRT-PCR confirmed that FGF19 mRNA levels were higher in NPC tissues (Fig. 1C). These results indicated that FGF19 is overexpressed in NPC patients.

### Serum FGF19 serves as a potential biomarker for NPC

As FGF19 can be secreted into serum and function as an endocrine factor, we then used ELISA to measure serum FGF19 levels. In the serum samples from 61 NPC patients, FGF19 ranged from 44.7 pg/mL to 984.7 pg/mL, while in 36 healthy volunteers, it ranged from 31.3 pg/mL to 494.9 pg/mL. NPC patients showed higher serum FGF19 levels (*P < 0.05) (Fig. 1D). Then, we analysed the association of serum FGF19 levels with patients’ clinical characteristics. The results showed that FGF19 levels were higher in patients in stages III- IV than in patients in stages I- II (*P < 0.05) (Fig. 1E), which was in accordance with the observation from the IHC analysis. However, it was not associated with patient age or sex (*P* > 0.05) (Fig. 1F-G). The above results indicated that FGF19 levels were increased in the serum of NPC patients and may act as a noninvasive novel biomarker.

### FGF19 regulates NPC cells malignant behaviours

**Fig. 2:**
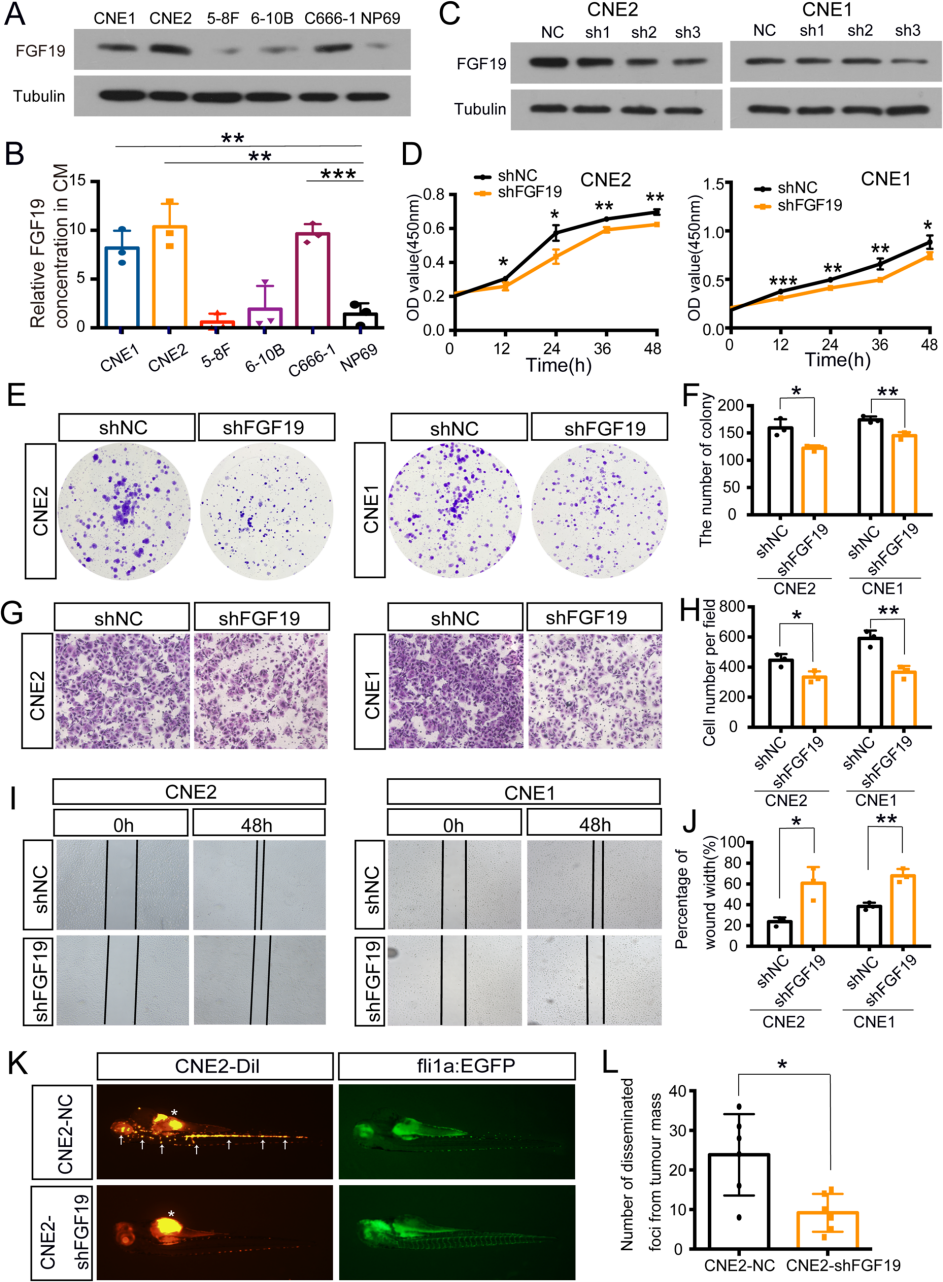
FGF19 regulates NPC cell malignant behaviours. A: Western blot analysis of FGF19 expression in NPC cell lines (CNE1, CNE2, 5-8F, 6-10B, C666-1) and the immortalized normal nasopharyngeal epithelial cell line NP69. B: ELISA was used to detect FGF19 level in culture medium(CM) of different cells. The column showed FGF19 concentration in CM from NPC cells relative to CM from NP69. C: The interference efficiency of shFGF19 was assessed by western blotting in CNE2 and CNE1 cells. D: CCK8 assay was used to determine cell proliferation after transfection with shNC or shFGF19 in CNE2 and CNE1 cells. E, F: Colony formation assay was performed in shNC or shFGF19 cells. We showed the representative images and the quantification analysis. G, H: Transwell assay was used to determine cell migration in shNC or shFGF19 cells. We showed the representative images and the quantification analysis. I, J: Wound healing assay was performed in shNC or shFGF19 cells. Representative images of cell migration were captured at 0 and 48 h with a microscope. The relative migrated width was calculated by the wound width/the distance measured at 0 h. The histogram showed the relative distance of wound. K: *Tg (fli1a: EGFP)* transgenic zebrafish were used to evaluate cell metastasis. shNC or shFGF19 CNE2 cells were stained with Dil and injected into the perivitelline cavity of zebrafish at 48 hpf. The migration of tumour cells was evaluated 2 days postinjection. The arrow represented the disseminated foci. We observed the disseminated foci from primary sites under a fluorescence microscope. Data are presented as the mean ± SD of three independent assessments. *P < 0.05, **P < 0.01, ***P < 0.001

Then, we investigated the role of FGF19 on NPC malignant behaviours. Western blotting demonstrated that FGF19 was more highly expressed in NPC cell lines (CNE1, CNE2, C666-1) than in NP69 cells (Fig. 2A). We also used ELISA to measure FGF19 levels in different cell culture supernatant. Among NPC cells, CNE2, CNE1 and C666-1 secreted higher levels of FGF19 than other cell lines (Fig. 2B). As FGF19 was highly expressed in CNE1, CNE2 and C666-1, we downregulated FGF19 in these cells. First, western blotting confirmed the knockdown efficiency (Fig. 2C, Figure S1A). CCK8 and colony formation assays showed that when FGF19 was knocked down, cell proliferation was inhibited (Fig. 2D-F, Figure S1B). Next, Transwell and wound healing assays showed that cell migration was inhibited in the shFGF19 group (Fig. 2G-J, Figure S1D,F). Additionally, to determine whether the ectopic expression of FGF19 could promote malignant properties, we transfected 5-8F with ectopic FGF19 as it had the lowest level of FGF19. Not surprisingly, cell proliferation and migration were enhanced in 5-8F oeFGF19 group (Figure S1A-G). Finally, we established a zebrafish migration model. shFGF19 or shNC CNE2 cells were injected into the perivitelline cavity of *Tg (fli1a: EGFP)* zebrafish. As shown in Fig. 2K-L, there were fewer disseminated tumour cells in zebrafish in the shFGF19 group than in the shNC group. Therefore, high expression of FGF19 could promote malignant NPC behaviour.

### FGF19 promotes NPC growth and positively correlates with MVD in vivo

**Fig. 3:**
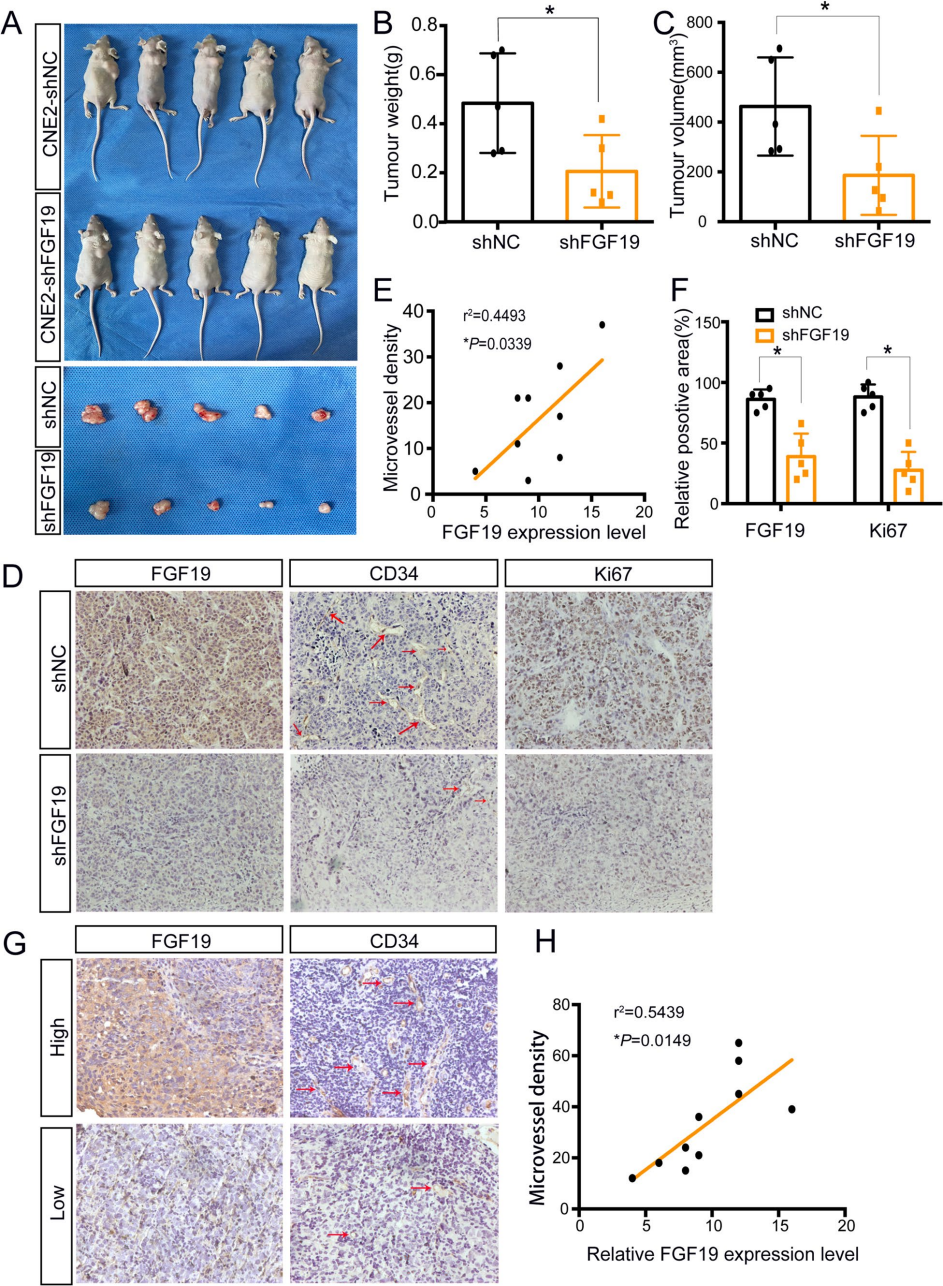
FGF19 promotes NPC growth and positively correlates with MVD in vivo. A: CNE2 cells transfected with shNC or shFGF19 were subcutaneously injected into nude mice. Representative pictures of NPC xenografts in nude mice are shown. B: The weights of the excised xenografts in the two groups. C: The volumes of the excised xenografts in the two groups. D: Representative results of immunohistochemical staining of FGF19, CD34 and Ki67 in xenograft sections. Red arrows indicate microvessels. E: Spearman correlation between FGF19 expression and MVD in tumour xenografts. The Pearson correlation coefficient (r^2^) and *P* value are shown. F: The column shows relative positive areas of FGF19 and Ki67 in xenografts according to the IHC results. G: Representative results of high and low immunohistochemical staining of FGF19 and CD34 in NPC tissues. Red arrows indicate microvessels. H: Spearman correlation between FGF19 expression and MVD in 10 NPC tissues. Pearson correlation coefficient (r^2^) and* P* value are shown. Data are presented as the mean ± SD of three independent assessments. *P < 0.05, **P < 0.01, ***P < 0.001

Next, we performed in vivo research to further confirm the role of FGF19. The results showed that tumour weight and volume of the mice were decreased in shFGF19-CNE2 or shFGF19-C666-1 group (Fig. 3A-C, Figure S2A-C). IHC revealed that when FGF19 was downregulated, Ki67 expression levels were suppressed, which indicated that FGF19 promotes NPC growth (Fig. 3D, F, Figure S2D, F). As the FGF family was reported to promote angiogenesis, we also investigated the function of FGF19 in NPC angiogenesis. Microvessel density (MVD) is widely used to evaluate angiogenesis and could act as a prognostic indicator in NPC [34]. As shown in Fig. 3D-E and Figure S2D-E, shFGF19 xenografts had decreased MVD and FGF19 expression was positively related to MVD. Thus, FGF19 promoted NPC growth and was related to angiogenesis.

Then, IHC was performed on 10 NPC tissues. As Fig. 3G-H illustrates, FGF19 expression was positively correlated with MVD, and tissues with higher FGF19 expression levels had increased MVD. Therefore, FGF19 is associated with NPC angiogenesis.

### NPC cells secrete FGF19 to HUVECs and promote angiogenesis

**Fig. 4:**
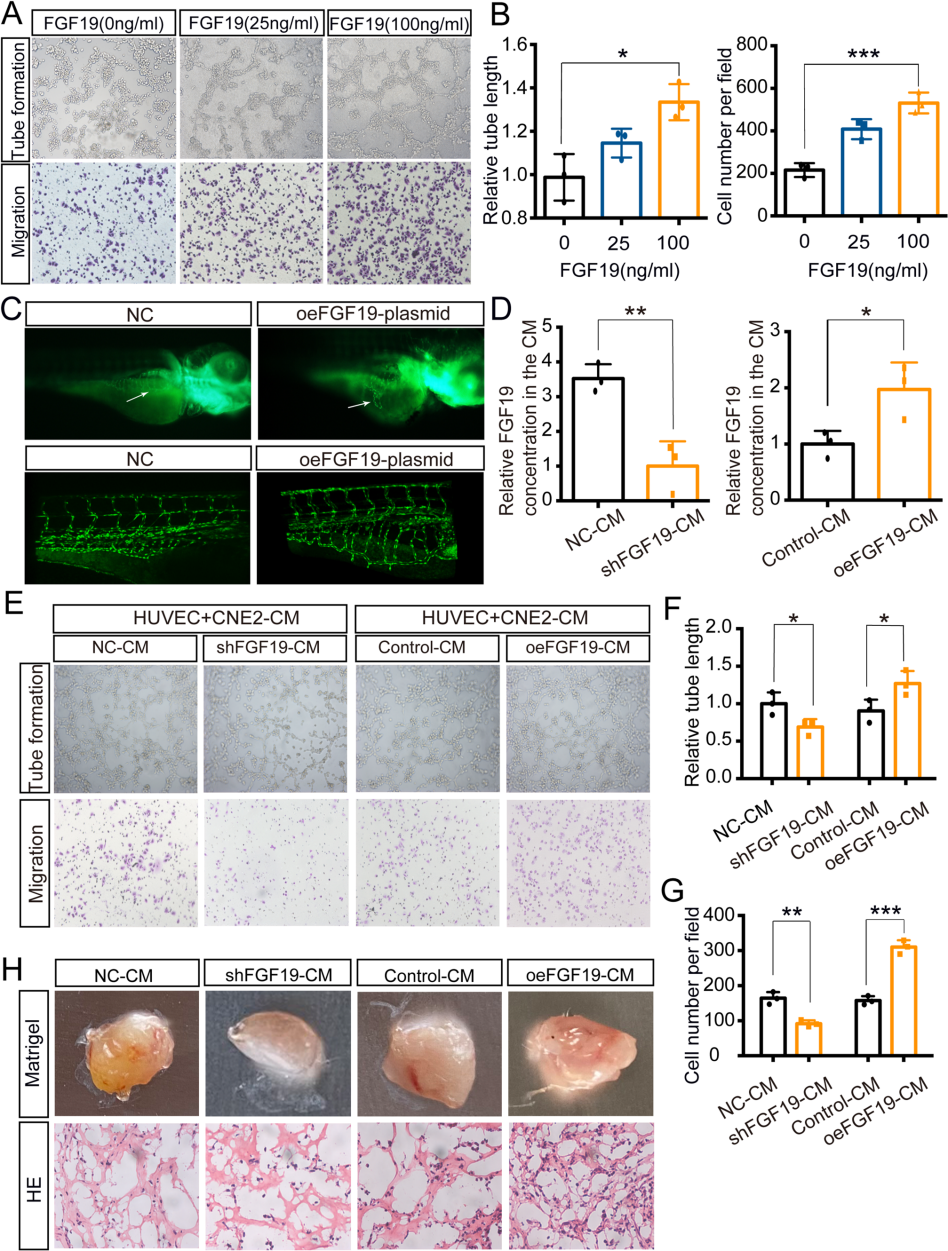
NPC cells secrete FGF19 into HUVECs and promote angiogenesis. A: Tube formation assays (top) and Transwell migration assays (bottom) were performed to measure tube formation and migration of HUVECs treated with increasing doses of FGF19. B: The relative tube length and number of migrated HUVECs were quantified. C: Morphology of subintestinal vessels (SIVs) in *Tg (fli1a: EGFP)* transgenic zebrafish after the injection of FGF19 plasmid or negative control. Up: Morphology of subintestinal vessels (SIVs) was photographed by a fluorescence microscope. Bottom: Morphology of SIVs was photographed by a confocal microscope. Arrows: sprouts of SIVs. D: Relative FGF19 level in culture medium(CM) collected from CNE2 cells transfected with shFGF19 or oeFGF19 plasmids. E: Tube formation assays (top) and Transwell migration assays (bottom) were performed to measure tube formation and migration of HUVECs treated with different CMs. F, G: The relative tube length and number of migrated HUVECs were quantified. H: HUVECs pretreated with different CMs were mixed with Matrigel for subcutaneous injection. Top: Gross observation of angiogenesis in Matrigel plugs. Bottom: H&E staining was performed to observe blood vessel formation in different groups. Data represent the mean ± SD of three independent experiments. *P < 0.05, **P < 0.01, ***P < 0.001

Since FGF19 is associated with MVD in NPC, we next focused on its role in angiogenesis. To evaluate whether FGF19 directly influences angiogenesis, we treated HUVECs with exogenous FGF19. With the increasing doses of FGF19, tube formation and migration of HUVECs were accelerated (Fig. 4A-B). *Tg (fli1a: EGFP)* transgenic zebrafish were used to observe vessel phenotypes. The results showed that FGF19 significantly promotes the sprouting of subintestinal vessels (SIVs) in zebrafish, which further confirmed the proangiogenic function of FGF19 (Fig. 4C).

Next, we collected culture medium (CM) from shFGF19- or oeFGF19-CNE2 cells and further identified the role of secreted FGF19 from NPC cells (Fig. 4D). We observed that shFGF19-CM inhibited the tube formation and migration of HUVECs, while oeFGF19-CM had the opposite effects (Fig. 4E-G). Moreover, the Matrigel plug angiogenesis assay showed that HUVECs pretreated with shFGF19-CM exhibited fewer vessels, while oeFGF19-CM-treated cells had more vessels (Fig. 4H). Additionally, we collected CM from shFGF19-C666-1 and oeFGF19-5-8F cells (Figure S3A), and the results further confirmed the promotion role of FGF19 on HUVECs (Figure S3B-D). Collectively, FGF19 derived from NPC cells promoted HUVECs angiogenesis.

### FGF19 upregulates ANXA2 expression to accelerate angiogenesis

**Fig. 5:**
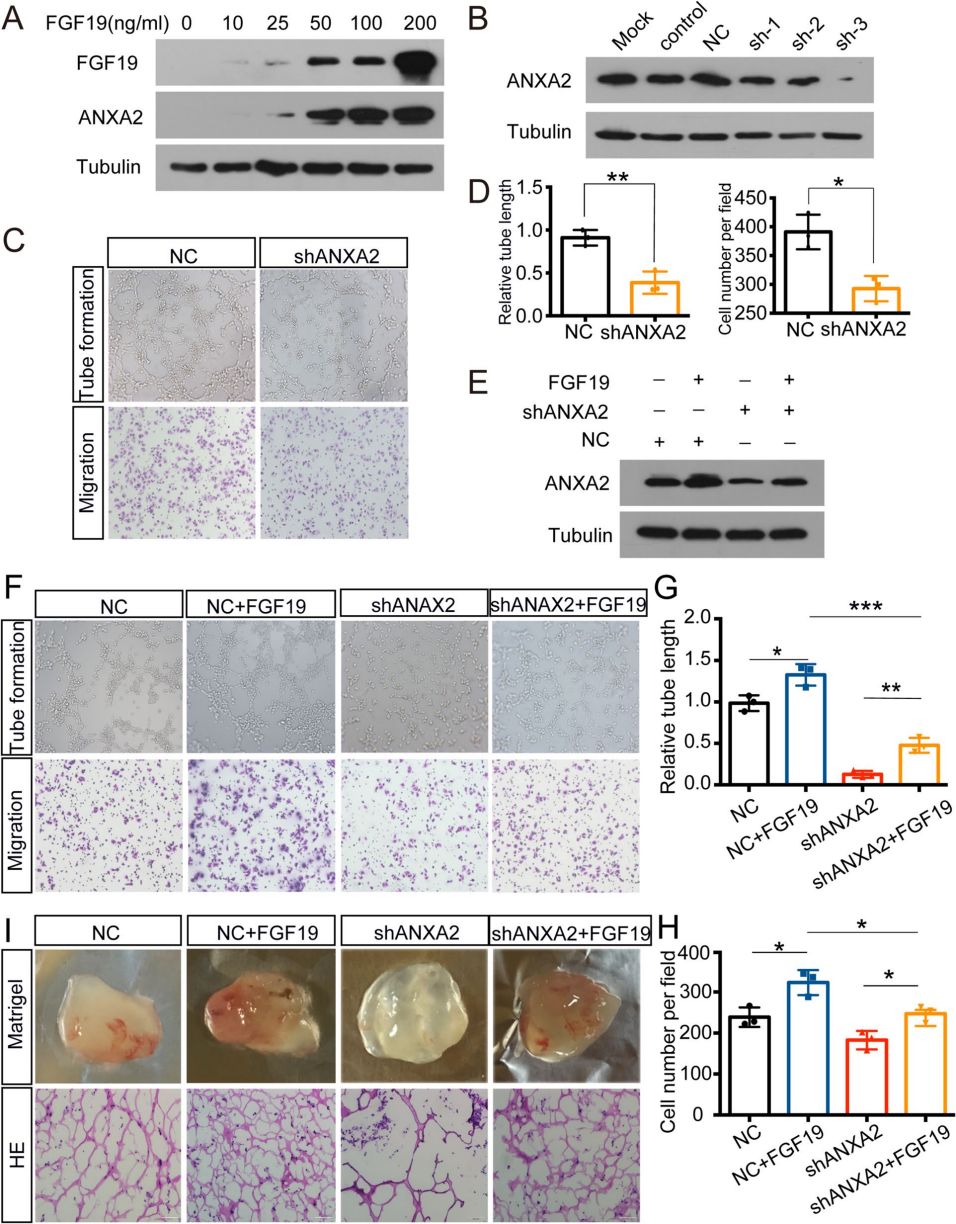
FGF19 influences ANXA2 expression to accelerate angiogenesis. A: ANXA2 expression in HUVECs with the increasing doses of FGF19. B: The interference efficiency of shANXA2 was assessed by western blotting in HUVECs. C, D: Tube formation assays (top) and Transwell migration assays (bottom) were performed to measure tube formation and migration of HUVECs transfected with shNC or shANXA2. E: Western blotting was used to detect ANXA2 expression with the treatment of FGF19 or shANXA2. F: Tube formation assays (top) and Transwell migration assays (bottom) were performed to measure tube formation and migration of HUVECs. G, H: The relative tube length and migrated HUVECs were quantified. I: HUVECs transfected with shANXA2 or shNC and pretreated with FGF19 were mixed with Matrigel for subcutaneous injection. Top: Gross observation of angiogenesis in Matrigel plugs. Bottom: H&E staining was performed to observe blood vessel formation in different groups. Data represent the mean ± SD of three independent experiments. *P < 0.05, **P < 0.01, ***P < 0.001

Then, we wondered how FGF19 promotes angiogenesis and its probable mechanism. Previous studies have confirmed that ANXA2 influences tumour angiogenesis [35], and we then determined ANXA2 expression after FGF19 treatment. With the increasing doses of FGF19, ANXA2 levels in HUVECs were elevated (Fig. 5A). Figure 5B-D confirmed that when ANXA2 was downregulated, tube formation and migration were inhibited. More importantly, western blotting showed the variation in ANXA2 with FGF19 treatment (Fig. 5E). Next, we tested whether ANXA2 is involved in the positive effect of FGF19 on HUVECs. The results showed that tube formation and migration were suppressed when ANXA2 was downregulated (Fig. 5F-H). The Matrigel plug angiogenesis assay also exhibited the similar results (Fig. 5I). Thus, the promoting function of FGF19 on angiogenesis relied on ANXA2 expression.

### TRIM21 interacts with ANXA2 and triggers ubiquitination

**Fig. 6:**
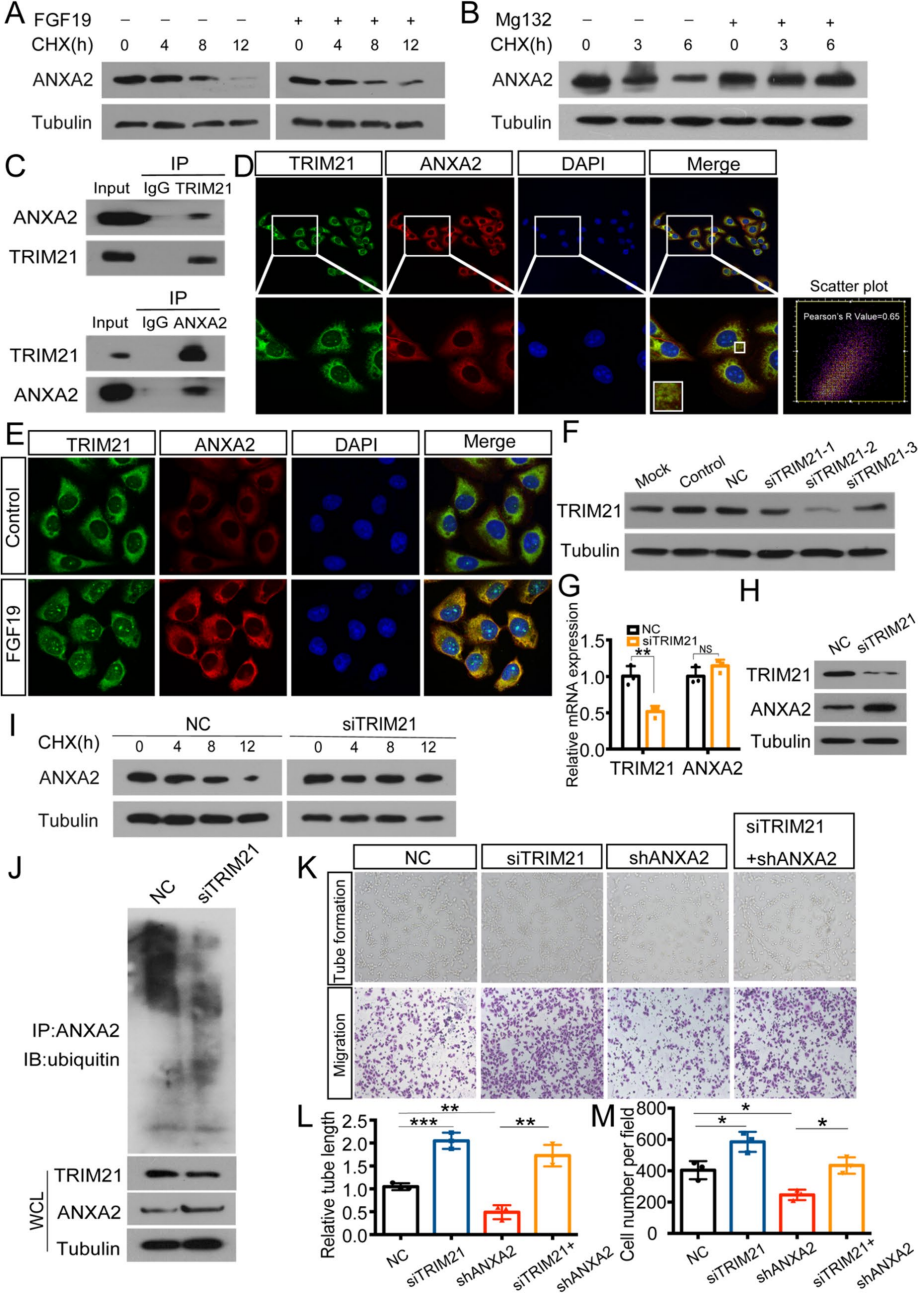
TRIM21 interacts with ANXA2 and triggers ubiquitination. A: ANXA2 expression in HUVECs with or without FGF19 treatment following CHX treatment for the indicated times. B: ANXA2 expression in HUVECs with or without the addition of Mg132 following CHX treatment for the indicated times. C: Co-IP was performed to analyse the interaction between ANXA2 and TRIM21 in HUVECs. IgG was used as a negative control. D: Representative images of colocalization of ANXA2 and TRIM21 in HUVECs. Red: ANXA2; Green: TRIM21. Pearson's R value of scatter plot analysis was calculated using ImageJ. E: ANXA2 and TRIM21 expression in HUVECs after FGF19 treatment was detected by immunofluorescence staining. F: The interference efficiency of siTRIM21 was assessed by western blotting in HUVECs. G: qRT-PCR was used to detect mRNA level of TRIM21 and ANXA2 with the transfection of NC or siTRIM21. H: Western blot was used to detect protein level of TRIM21 and ANXA2 with the transfection of NC or siTRIM21. I: ANXA2 expression in HUVECs transfected with NC or siTRIM21 following CHX treatment for the indicated times. J: HUVECs transfected with NC or siTRIM21 were immunoprecipitated with ANXA2 antibody and analysed by immunoblotting with the anti-ubiquitin antibody to examine ANXA2 ubiquitination. Whole-cell lysates were used for western blotting with an anti-TRIM21 or anti-ANXA2 antibody. K: Tube formation assays (top) and Transwell migration assays (bottom) were performed to measure tube formation and migration of shANXA2-HUVECs transfected with NC or siTRIM21. L, M: The relative tube length and number of migrated HUVECs were quantified. Data represent the mean ± SD of three independent experiments. *P < 0.05, **P < 0.01, ***P < 0.001

Since FGF19 influences ANXA2 expression, we then determined the effects of FGF19 on ANXA2 stability in HUVECs. Figure 6A showed that ANXA2 degradation was significantly delayed with FGF19 stimulation in the presence of the protein synthesis inhibitor cycloheximide (CHX), which suggested that FGF19 prevented the degradation of ANXA2. The ubiquitin/proteasome system is one of the most important processes for protein degradation. We treated cells with Mg132, which is a specific ubiquitin/proteasome inhibitor and found that it inhibited ANXA2 degradation (Fig. 6B). Therefore, ANXA2 degradation was associated with ubiquitin–proteasome and the influence of FGF19 on ANXA2 expression might rely on this way.

Next, we wondered how FGF19 influences ANXA2 ubiquitination. E3 ubiquitin ligase plays important roles in the ubiquitin/proteasome pathway. Since FGF19 is not an E3 ligase, we performed co-IP to identify the protein that had E3 ubiquitin ligase activity and could interact with ANXA2. From the co-IP results, TRIM21 interacted with ANXA2 in HUVECs (Fig. 6C). Moreover, the localization of ANXA2 and TRIM21 was observed by immunofluorescent colocalization analysis (Fig. 6D). Then, we also found that FGF19 greatly increased the expression of ANXA2 and decreased TRIM21 by immunofluorescence staining (Fig. 6E). Accordingly, we hypothesized that TRIM21 might be responsible for ANXA2 ubiquitination. Not surprisingly, siTRIM21 increased the protein level of ANXA2, while the mRNA level was not affected (Fig. 6F-H). Furthermore, HUVECs transfected with siTRIM21 or siNC were treated with CHX, and ANXA2 degradation was inhibited in siTRIM21-treated cells (Fig. 6I). Ubiquitination assays showed that when TRIM21 was downregulated, ANXA2 ubiquitin levels were decreased (Fig. 6J). To further elucidate the functional role of TRIM21, shANXA2-HUVECs were transfected with siTRIM21, which reversed the inhibitory effect of shANXA2 on HUVECs (Fig. 6K-M). Therefore, TRIM21 interacted with ANXA2 and could trigger its ubiquitination in HUVECs.

### NPC-derived FGF19 upregulates ANXA2 by blocking TRIM21-mediated ubiquitination

**Fig. 7:**
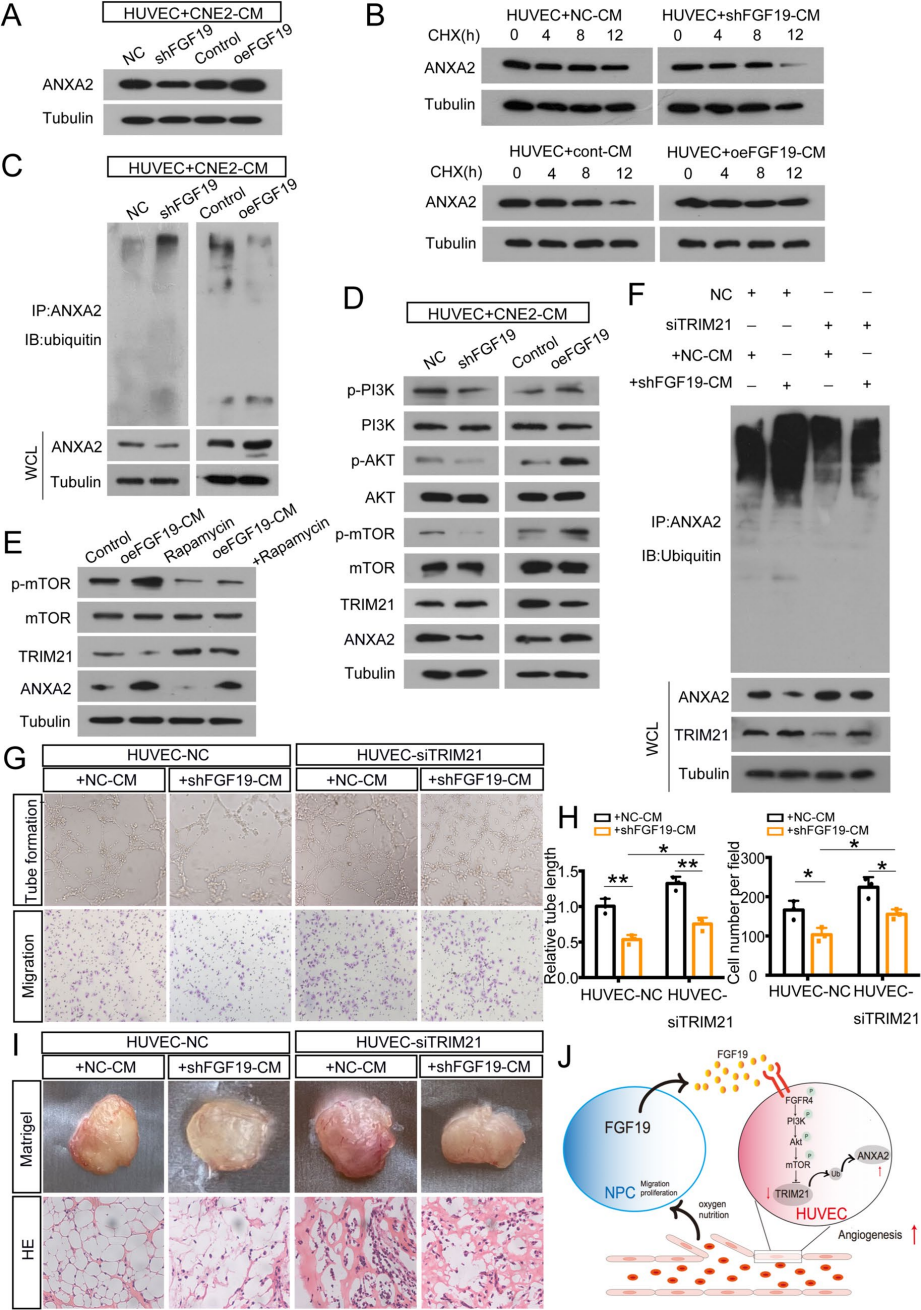
NPC-derived FGF19 upregulates ANXA2 by blocking TRIM21-mediated ubiquitination. A: ANXA2 expression in HUVECs treated with shFGF19-CM or oeFGF19-CM. B: ANXA2 expression in HUVECs treated with shFGF19-CM or oeFGF19-CM following CHX treatment for the indicated times. C: HUVECs treated with shFGF19-CM or oeFGF19-CM were immunoprecipitated with ANXA2 antibody and analysed by immunoblotting with the anti-ubiquitin antibody to examine ANXA2 ubiquitination. D: Western blot analysis of PI3K/AKT/mTOR in HUVECs treated with shFGF19-CM or oeFGF19-CM. E: Western blot analysis of p-mTOR in HUVECs with the treatment of oeFGF19-CM or the addition of rapamycin. F: HUVECs were transfected with siTRIM21 and treated with shFGF19-CM or NC-CM. Then cells were immunoprecipitated with ANXA2 antibody and analyzed by immunoblotting with the anti-ubiquitin antibody to examine ANXA2 ubiquitination. G: Tube formation assays (top) and Transwell migration assays (bottom) were performed to measure tube formation and migration of HUVECs pretransfected with siTRIM21 and cocultured with shFGF19-CM or NC-CM. H: The relative tube length and number of migrated HUVECs were quantified. I: HUVECs transfected with siTRIM21 or NC and cocultured with shFGF19-CM or NC-CM were mixed with Matrigel for subcutaneous injection. Top: Gross observation of angiogenesis in Matrigel plugs. Bottom: H&E staining was performed to observe blood vessel formation in different groups. J: A working model of FGF19 promoting NPC angiogenesis by influencing TRIM21-mediated ANXA2 ubiquitination through the activation of the PI3K/Akt/mTOR pathway. Data represent the mean ± SD of three independent experiments. *P < 0.05, **P < 0.01, ***P < 0.001

Furthermore, we investigated whether NPC-derived FGF19 could regulate ANXA2 ubiquitination. CMs from shFGF19- or oeFGF19-CNE2 were cocultured with HUVECs. Western blot analysis showed that ANXA2 expression was downregulated by shFGF19-CM treatment but upregulated by oeFGF19-CM treatment (Fig. 7A). We also collected CMs from shFGF19-C666-1 and oeFGF19-5-8F, ANXA2 showed a similar expression variation (Figure S4A). Then, we determined the effects of NPC-derived FGF19 on ANXA2 stability. As shown in Fig. 7B, ANXA2 degradation was delayed with oeFGF19-CM treatment. Ubiquitination assays confirmed that oeFGF19-CM downregulated ANXA2 polyubiquitination level, and shFGF19-CM increased ANXA2 polyubiquitination (Fig. 7C). HUVECs treated with shFGF19-C666-1 or oeFGF19-5-8F also influence ANXA2 polyubiquitination level (Figure S4B-C). These results suggested that NPC-derived FGF19 prevented ANXA2 ubiquitination and degradation.

The PI3K/AKT pathway was reported to regulate angiogenesis and could contribute to abnormal blood vessel formation [36]. We found that p-PI3K, p-AKT1 and p-mTOR were elevated with oeFGF19-CM treatment, while shFGF19-CM had the opposite effect (Fig. 7D, Figure S4D). Moreover, we found that mTOR activity was inhibited when cells were treated with rapamycin, which is a well-known mTOR inhibitor [37]. The inhibitory effect was reversed with oeFGF19-CM treatment (Fig. 7E, Figure S4E). Therefore, the promotion of angiogenesis by FGF19 might be associated with the PI3K/AKT/mTOR pathway.

Furthermore, we wondered whether NPC-derived FGF19 could regulate ANXA2 expression by influencing TRIM21-mediated ubiquitination. From the immunoprecipitation assay, we observed that with shFGF19-CM treatment, ubiquitination of ANXA2 was increased, while when HUVECs were pretransfected with siTRIM21, ANXA2 ubiquitination was decreased (Fig. 7F).

Finally, we elucidated the functional roles in vitro. HUVECs were pretransfected with siTRIM21, and subsequently treated with shFGF19-CM from CNE2 or C666-1. The results showed that shFGF19-CM decreased tube formation and cell migration, while cells transfected with siTRIM21 could partly reverse this effect (Fig. 7G-I, Figure S4F-I). Altogether, NPC-derived FGF19 regulated angiogenesis by influencing TRIM21-mediated ANXA2 ubiquitination.

## Discussion

Several studies have focused on the molecular landscape of NPC and identified multiple factors that contribute to NPC progression. However, its in-depth mechanism remains to be further explored. In this research, we found that high expression of FGF19 could promote NPC progression. We then investigated the role of FGF19 in angiogenesis and identified that NPC cells secreted high levels of FGF19, promoting the angiogenesis of HUVECs. Mechanistically, FGF19 regulated angiogenesis by influencing TRIM21-mediated ANXA2 ubiquitination through PI3K/Akt/mTOR (Fig. 7J). This evidence demonstrates the importance of FGF19 in NPC progression.

FGF19, belongs to the endocrine FGF family, acts as a signalling molecule and is overexpressed in a subgroup of tumours [38]. However, the role of FGF19 in NPC remains unclear. One of the most important findings of this research was that FGF19 is highly expressed in NPC tissues and associated with clinical stages (Fig. 1A-C). In a previous study on head and neck squamous cell carcinoma(HNSCC), FGF19 was overexpressed in tumours and promoted cell proliferation [31]. In our study, we confirmed that FGF19 accelerates NPC cell malignant behaviours (Figs. 2 and 3, Figure S1-2).

As an endocrine factor, FGF19 diffuses into circulation to drive interorgan crosstalk [39]. The concentration of FGF19 in circulation is associated with the pathogenesis of diseases. In hepatocellular carcinoma(HCC) and melanoma, serum FGF19 levels were significantly elevated in tumour patients [40, 41]. In accordance with these studies, we found that FGF19 levels were higher in the serum of NPC patients and had a close relationship with tumour stage, supporting the importance of FGF19 in NPC diagnosis (Fig. 1D-E). In HNSCC, secreted FGF19 levels were correlated with FGF19 expression in tumours [31]. We also evaluated FGF19 expression in NPC cell lines. Not surprisingly, it was elevated in the culture medium of CNE2, CNE1 and C666-1 cells, which was consistent with the higher expression of protein levels in NPC cells (Fig. 2B). Therefore, FGF19 could be easily detected and might serve as a noninvasive biomarker for NPC.

Abnormal tumour vasculature might have profound consequences for tumour survival. Tumour and stromal cells secrete several growth factors to promote abnormal tumour vessel formation. In colorectal cancer, secreted dickkopf2 might be a potential antiangiogenic target for patients [42]. FGFs can also be secreted and induce angiogenic responses. FGF2 was highly expressed in antiangiogenic drug-resistant NPC, and its knockdown reduced tumour angiogenesis [43]. Here, we identified a previously unreported function of FGF19 in angiogenesis. We found that FGF19 overexpression was accompanied by MVD in both clinical NPC tissues and subcutaneous tumours (Fig. 3D-E, G-H, Figure S2D-E). Moreover, HUVECs cocultured with oeFGF19-CM exhibited more tubes and vessels (Fig. 4E-H, Figure S3B-D). Therefore, novel mechanisms by which FGF19 promotes angiogenesis need to be investigated in-depth.

FGF/FGFR is an important pathway that participates in tumour progression, including cell proliferation, migration, angiogenesis and immune evasion [39, 44]. Studies have confirmed that FGF19 binds exclusively to FGFR4. We previously identified that FGFR4, the receptor of FGF19, promoted NPC pathogenesis and might act as a prognostic biomarker [32]. Similar results were also reported in HCC; FGF19-FGFR4 mediated the upregulation of SOX8 and promoted HCC metastasis [45]. FGF19-FGFR4 signalling activates several pathways, including extracellular regulated protein kinases(ERK), jun N-terminal kinase (JNK), phosphoinositide 3-kinase (PI3K), mammalian target of rapamycin (mTOR) and others, which are closely related to tumour progression [28, 45, 46]. In HNSCC, FGF19 activated FGFR4-dependent ERK/AKT-p70S6K-S6 signalling to promote cell proliferation [31]. We also reported that FGF19 could activate FGF19-FGFR4-dependent ERK signaling cascade to exert its function [47]. Previous research has confirmed that HUVECs natively express FGFR4 [48], so we hypothesized that FGF19 binds to FGFR4 in HUVECs and activates downstream signals.

PI3K/AKT was reported to be activated by FGF19/FGFR4 and involved in tumour progression. Silencing FGF19 or neutralizing extracellular FGF19 with an anti-FGF19 antibody (1A6) decreased FGFR4-mediated AKT phosphorylation and inhibited cell growth, which further enhanced drug sensitivity [30]. PI3K/AKT could also regulate angiogenesis [36]. In our research, we found that FGF19 activates the PI3K/AKT1/mTOR pathway in HUVECs (Fig. 7D, Figure S4D). Moreover, we found rapamycin, a well-known mTOR inhibitor, could inhibit mTOR activity, and the inhibitory effect was reversed with oeFGF19-CM treatment (Fig. 7E, Figure S4E). Therefore, the promotion of angiogenesis by FGF19 might be associated with the PI3K/AKT1/mTOR pathway.

ANXA2, a 36 kDa protein, is involved in autoimmune and neurodegenerative diseases and malignant tumours [49]. It also activates factors that promote angiogenesis [50]. Silencing ANXA2 was able to suppress HUVEC proliferation and represented a useful target for future therapies [51]. In breast cancer, blocking ANXA2 significantly inhibited neoangiogenesis [52]. We found that with the increasing of FGF19 levels, ANXA2 levels were also elevated, while when ANXA2 was downregulated, the promotion function of FGF19 was inhibited (Fig. 5E-I). Therefore, FGF19 promoted angiogenesis by influencing ANXA2 expression. Then, we wondered how FGF19 influences ANXA2 expression. Posttranslational modifications, such as ubiquitination, can regulate protein stability. FGF19 was reported to increase SHP stability by inhibiting its ubiquitination [53]. In this study, we found that FGF19 prevented ANXA2 degradation via the ubiquitin/proteasome pathway (Fig. 6A-B). Moreover, oeFGF19-CM downregulated ANXA2 polyubiquitination levels (Fig. 7B-C, Figure S4B-C). Therefore, NPC-derived FGF19 prevented ANXA2 ubiquitination and degradation.

TRIM21, a member of the TRIM family, can degrade proteins in proteasomes via E3 ubiquitin ligase activity [54]. As we mentioned, TRIM21 interacted with ANXA2 and inhibited its expression via the ubiquitin/proteasome system (Fig. 6C-J). In a study on colitis-associated cancer, TRIM21 was found to negatively modulate epithelial cell proliferation and angiogenesis [55]. We observed that when TRIM21 was downregulated, tube formation was promoted (Fig. 6K-M). Previously, TRIM21 was reported as a target of PI3K/AKT signalling and this pathway negatively regulates its expression [56]. Consistently, in HUVECs, TRIM21 was downregulated followed by PI3K/AKT/mTOR activation with oeFGF19-CM treatment (Fig. 7D, Figure S4D). Moreover, siTRIM21 could partly reverse the inhibitory effect of shFGF19-CM on angiogenesis (Fig. 7F-I, Figure S4F-I).

## Conclusion

Altogether, we identified that FGF19, which was overexpressed in NPC, could enhance angiogenesis and benefit the NPC malignant phenotype. It accelerated angiogenesis by influencing TRIM21-mediated ANXA2 ubiquitination through the activation of PI3K/Akt/mTOR. Therefore, FGF19 might be a novel target for NPC diagnosis and therapy.

## Supplementary Information

Below is the link to the electronic supplementary material.

Supplementary file1 Figure Supplementary1. FGF19 regulates NPC cells malignant behaviours in C666-1 and 5-8F cells. A: The efficiency of shFGF19 or oeFGF19 was assessed by western blotting in C666-1 and 5-8F cells. B, C: Colony formation assay was performed in C666-1 and 5-8F cells. We showed the representative images and the quantification analysis. D, E: Transwell assay was used to determine cell migration in C666-1 and 5-8F cells. We showed the representative images and the quantification analysis. F, G: Wound healing assay was performed in C666-1 and 5-8F cells. Representative images of cell migration were captured at 0 and 48 h with a microscope. The relative migrated width was calculated by the wound width/the distance measured at 0 h. The histogram showed the relative distance of wound. Data are presented as the mean ± SD of three independent assessments. *P < 0.05, **P < 0.01, ***P < 0.001 (TIF 14626 KB)

Supplementary file2 Figure Supplementary2. FGF19 promotes C666-1 cells growth and positively correlates with MVD in vivo. A: C666-1 cells transfected with shNC or shFGF19 were subcutaneously injected into nude mice. Representative pictures of NPC xenografts in nude mice are shown. B: The weights of the excised xenografts in the two groups. C: The volumes of the excised xenografts in the two groups. D: Representative results of immunohistochemical staining of FGF19, CD34 and Ki67 in xenograft sections. Red arrows indicate microvessels. E: Spearman correlation between FGF19 expression and MVD in tumour xenografts. The Pearson correlation coefficient (r2) and *P* value were shown. F: The column shows relative positive areas of FGF19 and Ki67 in xenografts according to the IHC results. Data represent the mean ± SD of three independent experiments. *P < 0.05, **P < 0.01, ***P < 0.001 (TIF 17509 KB)

Supplementary file3 Figure Supplementary3. Secreted FGF19 from C666-1 and 5-8F cells could influence angiogenesis of HUVECs. A: Relative FGF19 level in culture medium(CM) collected from C666-1 cells transfected with shFGF19 or 5-8F cells transfected with oeFGF19 plasmids. B: Tube formation assays (top) and Transwell migration assays (bottom) were performed to measure tube formation and migration of HUVECs treated with different CMs. C: The relative tube length and number of migrated HUVECs were quantified. D: HUVECs pretreated with different CMs were mixed with Matrigel for subcutaneous injection. Data represent the mean ± SD of three independent experiments. *P < 0.05, **P < 0.01, ***P < 0.001 (TIF 13798 KB)

Supplementary file4 Figure Supplementary4. C666-1-derived FGF19 upregulates ANXA2 by blocking TRIM21-mediated ubiquitination. A: ANXA2 expression in HUVECs treated with shFGF19-CM from C666-1 or oeFGF19-CM from 5-8F. B: ANXA2 expression in HUVECs treated with shFGF19-CM from C666-1 or oeFGF19-CM from 5-8F following CHX treatment for the indicated times. C: HUVECs treated with shFGF19-CM or oeFGF19-CM were immunoprecipitated with ANXA2 antibody and analysed by immunoblotting with the anti-ubiquitin antibody to examine ANXA2 ubiquitination. D: Western blot analysis of PI3K/AKT/mTOR in HUVECs treated with shFGF19-CM from C666-1 or oeFGF19-CM from 5-8F. E: Western blot analysis of p-mTOR in HUVECs with the treatment of oeFGF19-CM from C666-1or the addition of rapamycin. F: Tube formation assays (top) and Transwell migration assays (bottom) were performed to measure tube formation and migration of HUVECs transfected with siTRIM21 and cocultured with shFGF19-CM from C666-1 or NC-CM. G, H: The relative tube length and number of migrated HUVECs were quantified. I: HUVECs transfected with siTRIM21 or NC and cocultured with shFGF19-CM from C666-1 or NC-CM were mixed with Matrigel for subcutaneous injection. Data represent the mean ± SD of three independent experiments. *P < 0.05, **P < 0.01, ***P < 0.001 (TIF 21554 KB)

## Data Availability

All data generated or analyzed during this study are included in this published article.

## Abbreviations

NPC: Nasopharyngeal carcinoma
FGF19: Fibroblast growth factor 19
FGF: Fibroblast growth factor
ANXA2: Annexin A2
TRIM21: Tripartite motif-containing 21
HUVECs: Human umbilical vein endothelial cells
IHC: Immunohistochemistry
MVD: Microvessel density
SIVs: Subintestinal vessels
CHX: Cycloheximide
CM: Culture medium
ERK: Extracellular regulated protein kinases
JNK: Jun N-terminal kinase
PI3K: Phosphoinositide 3-kinase
mTOR: Mammalian target of rapamycin
