# Supplementary figures and images for: FGF19 promotes nasopharyngeal carcinoma progression by inducing angiogenesis via inhibiting TRIM21-mediated ANXA2 ubiquitination
Source: Cell Oncol (Dordr). 2023 Oct 2;47(1):283–301. doi: 10.1007/s13402-023-00868-9 (PMC10899426; doi:10.1007/s13402-023-00868-9)

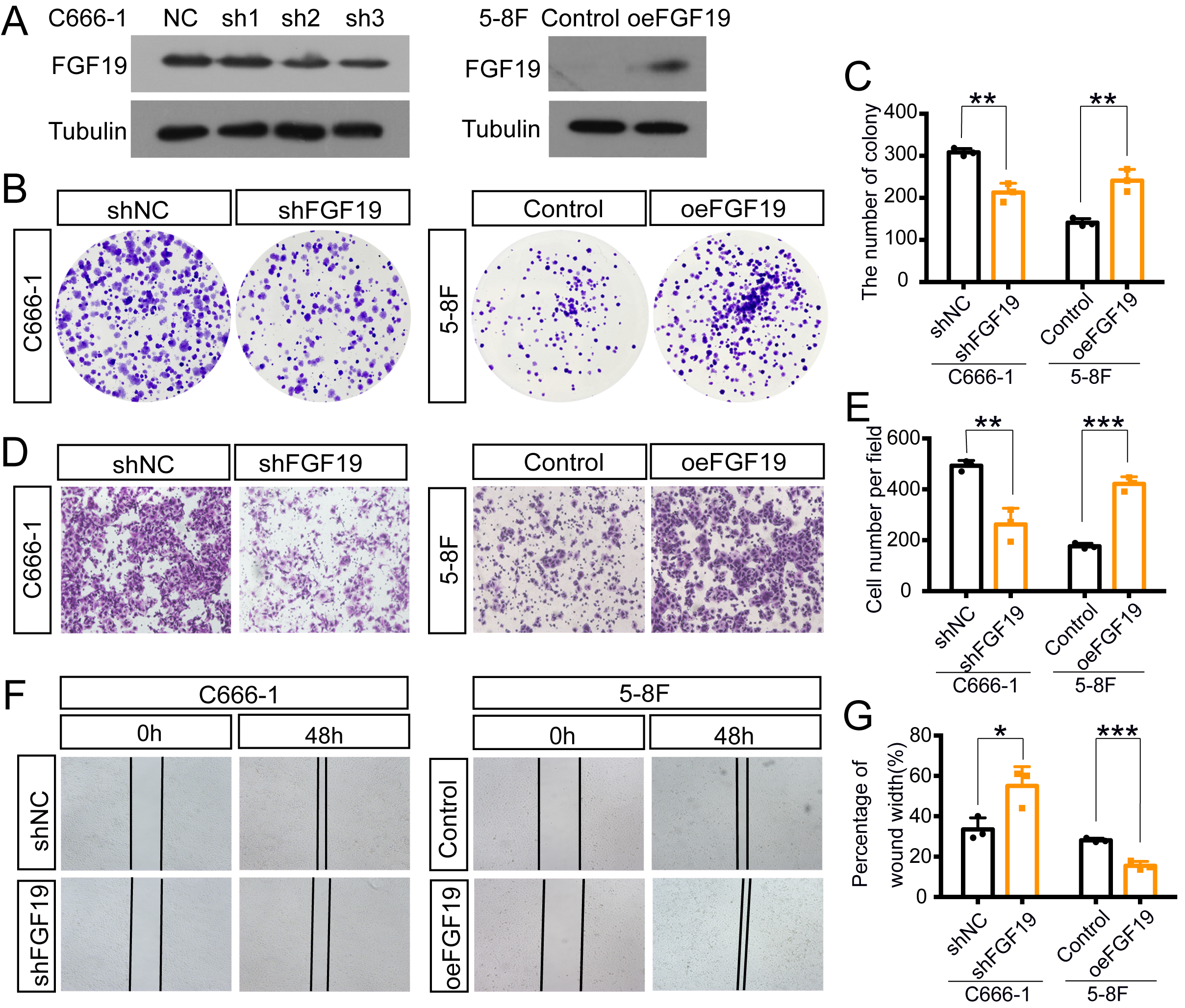

Supplement: Supplementary file 1 — Supplementary file1 Figure Supplementary1. FGF19 regulates NPC cells malignant behaviours in C666-1 and 5-8F cells. A: The efficiency of shFGF19 or oeFGF19 was assessed by western blotting in C666-1 and 5-8F cells. B, C: Colony formation assay was performed in C666-1 and 5-8F cells. We showed the representative images and the quantification analysis. D, E: Transwell assay was used to determine cell migration in C666-1 and 5-8F cells. We showed the representative images and the quantification analysis. F, G: Wound healing assay was performed in C666-1 and 5-8F cells. Representative images of cell migration were captured at 0 and 48 h with a microscope. The relative migrated width was calculated by the wound width/the distance measured at 0 h. The histogram showed the relative distance of wound. Data are presented as the mean ± SD of three independent assessments. *P < 0.05, **P < 0.01, ***P < 0.001 (TIF 14626 KB) [file 13402_2023_868_MOESM1_ESM.tif]

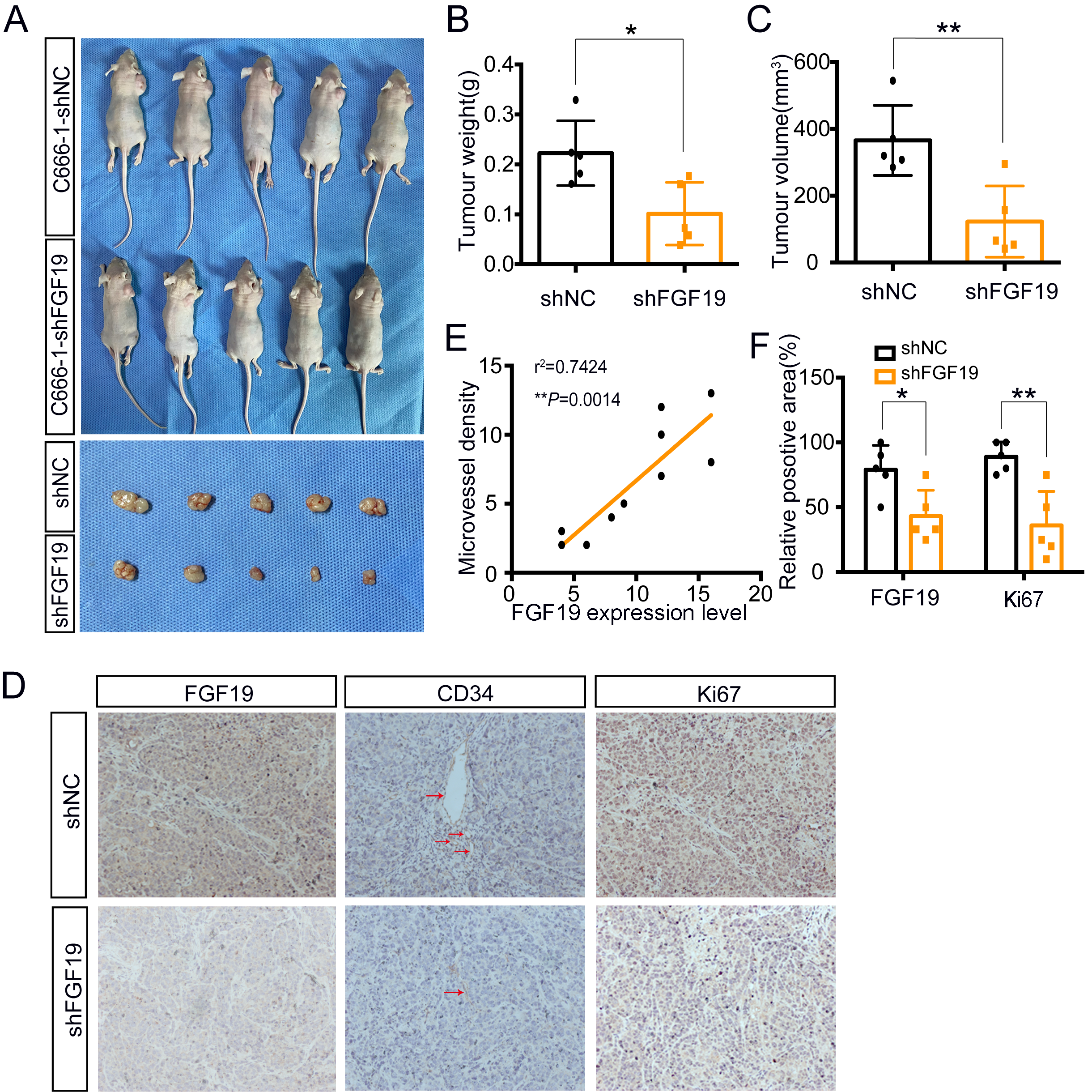

Supplement: Supplementary file 2 — Supplementary file2 Figure Supplementary2. FGF19 promotes C666-1 cells growth and positively correlates with MVD in vivo. A: C666-1 cells transfected with shNC or shFGF19 were subcutaneously injected into nude mice. Representative pictures of NPC xenografts in nude mice are shown. B: The weights of the excised xenografts in the two groups. C: The volumes of the excised xenografts in the two groups. D: Representative results of immunohistochemical staining of FGF19, CD34 and Ki67 in xenograft sections. Red arrows indicate microvessels. E: Spearman correlation between FGF19 expression and MVD in tumour xenografts. The Pearson correlation coefficient (r2) and P value were shown. F: The column shows relative positive areas of FGF19 and Ki67 in xenografts according to the IHC results. Data represent the mean ± SD of three independent experiments. *P < 0.05, **P < 0.01, ***P < 0.001 (TIF 17509 KB) [file 13402_2023_868_MOESM2_ESM.tif]

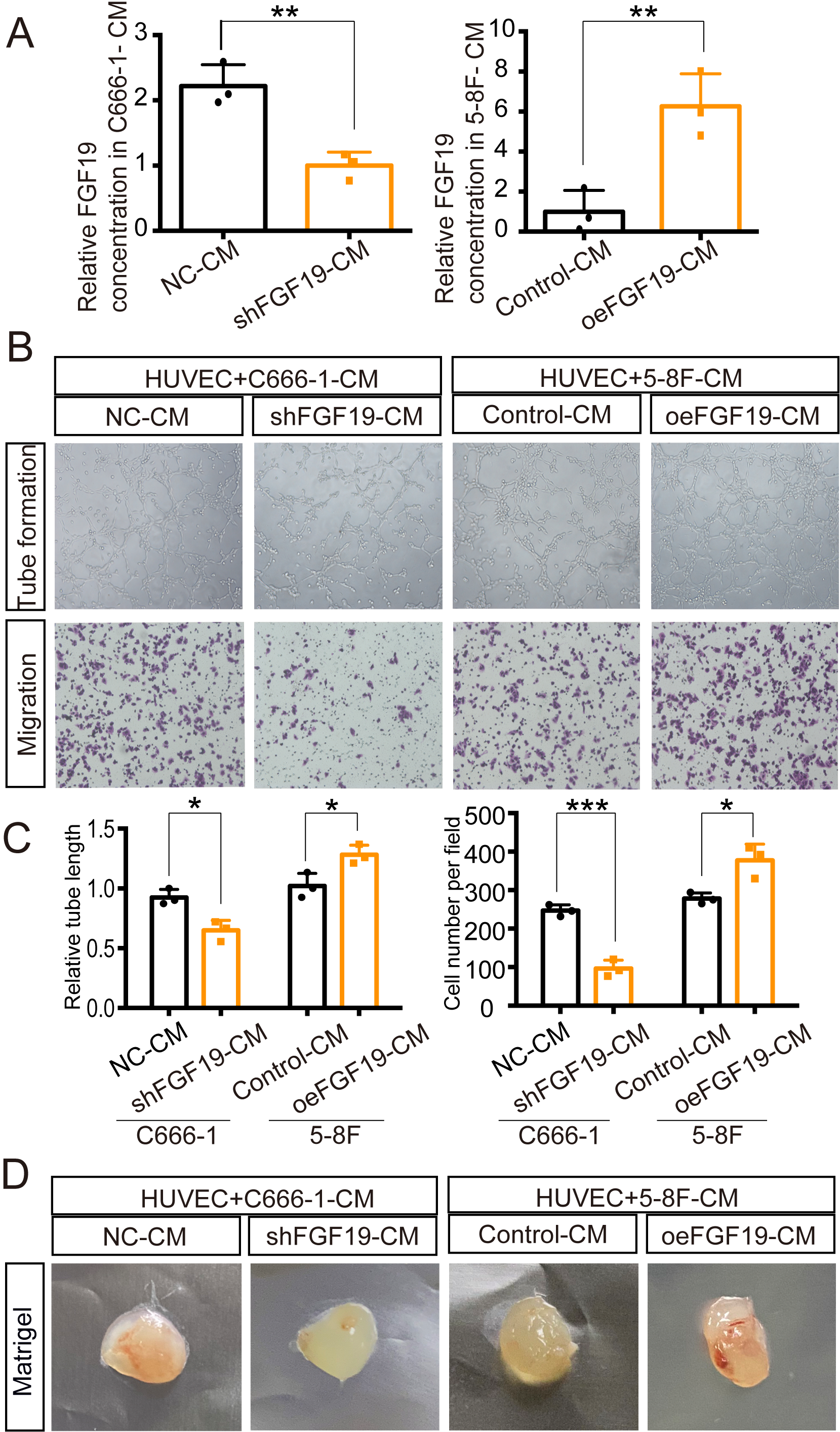

Supplement: Supplementary file 3 — Supplementary file3 Figure Supplementary3. Secreted FGF19 from C666-1 and 5-8F cells could influence angiogenesis of HUVECs. A: Relative FGF19 level in culture medium(CM) collected from C666-1 cells transfected with shFGF19 or 5-8F cells transfected with oeFGF19 plasmids. B: Tube formation assays (top) and Transwell migration assays (bottom) were performed to measure tube formation and migration of HUVECs treated with different CMs. C: The relative tube length and number of migrated HUVECs were quantified. D: HUVECs pretreated with different CMs were mixed with Matrigel for subcutaneous injection. Data represent the mean ± SD of three independent experiments. *P < 0.05, **P < 0.01, ***P < 0.001 (TIF 13798 KB) [file 13402_2023_868_MOESM3_ESM.tif]

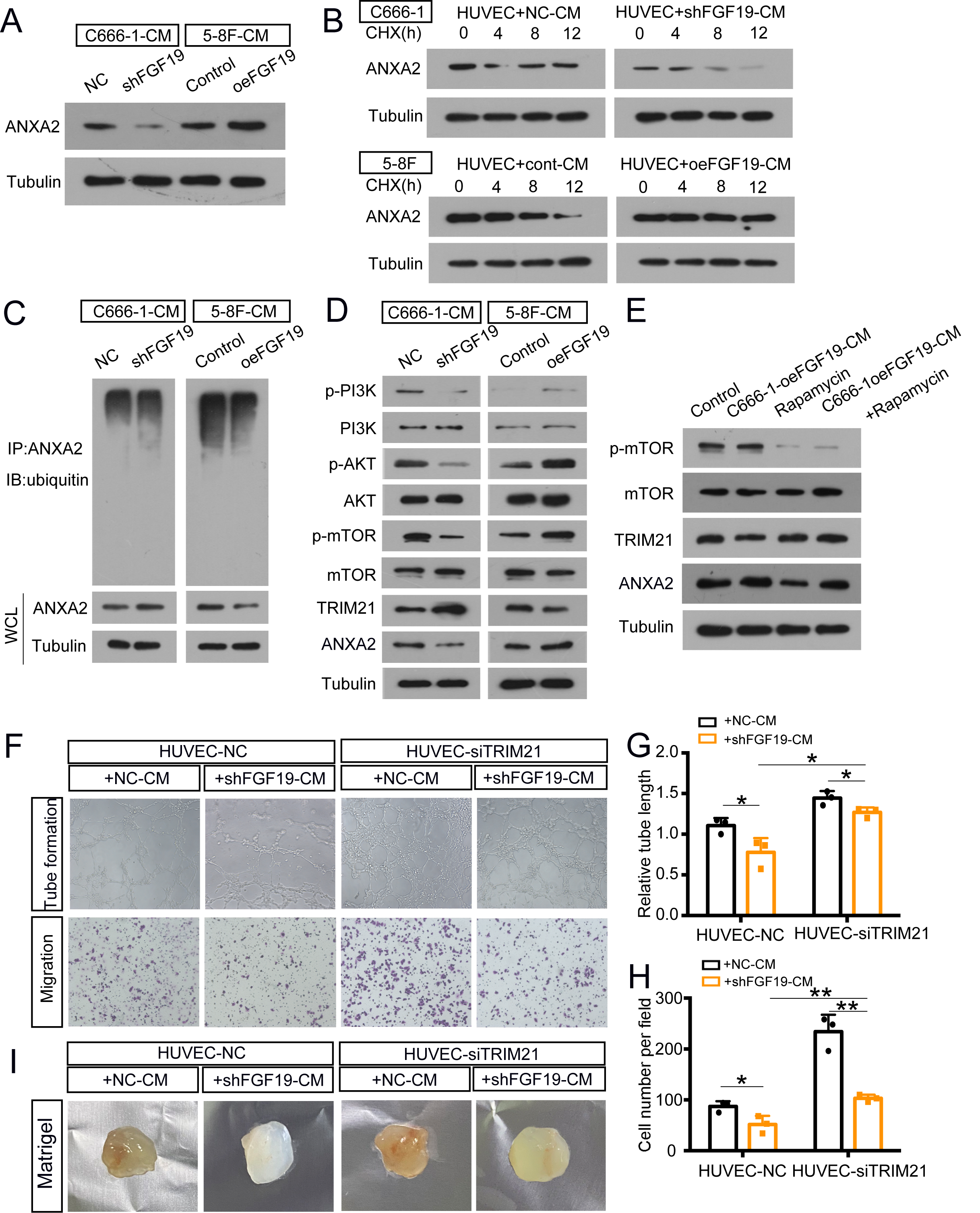

Supplement: Supplementary file 4 — Supplementary file4 Figure Supplementary4. C666-1-derived FGF19 upregulates ANXA2 by blocking TRIM21-mediated ubiquitination. A: ANXA2 expression in HUVECs treated with shFGF19-CM from C666-1 or oeFGF19-CM from 5-8F. B: ANXA2 expression in HUVECs treated with shFGF19-CM from C666-1 or oeFGF19-CM from 5-8F following CHX treatment for the indicated times. C: HUVECs treated with shFGF19-CM or oeFGF19-CM were immunoprecipitated with ANXA2 antibody and analysed by immunoblotting with the anti-ubiquitin antibody to examine ANXA2 ubiquitination. D: Western blot analysis of PI3K/AKT/mTOR in HUVECs treated with shFGF19-CM from C666-1 or oeFGF19-CM from 5-8F. E: Western blot analysis of p-mTOR in HUVECs with the treatment of oeFGF19-CM from C666-1or the addition of rapamycin. F: Tube formation assays (top) and Transwell migration assays (bottom) were performed to measure tube formation and migration of HUVECs transfected with siTRIM21 and cocultured with shFGF19-CM from C666-1 or NC-CM. G, H: The relative tube length and number of migrated HUVECs were quantified. I: HUVECs transfected with siTRIM21 or NC and cocultured with shFGF19-CM from C666-1 or NC-CM were mixed with Matrigel for subcutaneous injection. Data represent the mean ± SD of three independent experiments. *P < 0.05, **P < 0.01, ***P < 0.001 (TIF 21554 KB) [file 13402_2023_868_MOESM4_ESM.tif]
